# Cognitive and cognitive-motor interventions affecting physical functioning: A systematic review

**DOI:** 10.1186/1471-2318-11-29

**Published:** 2011-06-08

**Authors:** Giuseppe Pichierri, Peter Wolf, Kurt Murer, Eling D de Bruin

**Affiliations:** 1 Institute of Human Movement Sciences and Sport, ETH Zurich, Switzerland; 2 Department of Mechanical and Process Engineering, ETH Zurich, Switzerland

## Abstract

**Background:**

Several types of cognitive or combined cognitive-motor intervention types that might influence physical functions have been proposed in the past: training of dual-tasking abilities, and improving cognitive function through behavioral interventions or the use of computer games. The objective of this systematic review was to examine the literature regarding the use of cognitive and cognitive-motor interventions to improve physical functioning in older adults or people with neurological impairments that are similar to cognitive impairments seen in aging. The aim was to identify potentially promising methods that might be used in future intervention type studies for older adults.

**Methods:**

A systematic search was conducted for the Medline/Premedline, PsycINFO, CINAHL and EMBASE databases. The search was focused on older adults over the age of 65. To increase the number of articles for review, we also included those discussing adult patients with neurological impairments due to trauma, as these cognitive impairments are similar to those seen in the aging population. The search was restricted to English, German and French language literature without any limitation of publication date or restriction by study design. Cognitive or cognitive-motor interventions were defined as dual-tasking, virtual reality exercise, cognitive exercise, or a combination of these.

**Results:**

28 articles met our inclusion criteria. Three articles used an isolated cognitive rehabilitation intervention, seven articles used a dual-task intervention and 19 applied a computerized intervention. There is evidence to suggest that cognitive or motor-cognitive methods positively affects physical functioning, such as postural control, walking abilities and general functions of the upper and lower extremities, respectively. The majority of the included studies resulted in improvements of the assessed functional outcome measures.

**Conclusions:**

The current evidence on the effectiveness of cognitive or motor-cognitive interventions to improve physical functioning in older adults or people with neurological impairments is limited. The heterogeneity of the studies published so far does not allow defining the training methodology with the greatest effectiveness. This review nevertheless provides important foundational information in order to encourage further development of novel cognitive or cognitive-motor interventions, preferably with a randomized control design. Future research that aims to examine the relation between improvements in cognitive skills and the translation to better performance on selected physical tasks should explicitly take the relation between the cognitive and physical skills into account.

## Background

Age-related deteriorations in physical functioning have been attributed to decreases in sensory or motor system function [1]. Physical functioning refers to the ability to conduct a variety of activities ranging from self-care (instrumental activities of daily living) to more challenging mobility tasks that require balance abilities, strength or endurance, e.g. walking or standing, important for achieving or maintaining an independent way of living [2,3]. Until recently, for example, gait was considered an automated motor activity requiring minimal higher-level cognitive input [4]. Therefore, it seemed only logical that prevention of falls was mainly focused on exercises that address the modifiable physical aspects of fall related mobility impairments, e.g. strength and balance training [5-7]. Consistent evidence has been accumulated that regular physical training can improve muscle strength, aerobic capacity and balance, and delay the point in time when older adults need assistance to manage activities of daily living [5]. Maintenance of postural control during activities of daily living does not usually place high demands on attentional resources of healthy young or middle-aged people. In contrast, when sensory or motor deficits occur due to the natural aging process, the complex generation of movement may have to be adjusted. Movements may then be controlled and performed at an associative or a cognitive stage. Consequently, the postural control of older adults might be more vulnerable to cognitive distractions and additional tasks [8]. Recent research indicates that the influence of motor and sensory impairments on falls is in part moderated by the executive functions [9] and, thus, some of the causes of gait disturbances might also be attributed to changes in the executive functions [4], e.g., changes in divided attention [10,11]. Executive function refers to cognitive processes that control and integrate other cognitive activities [12,13], and this term has been used to describe a group of cognitive actions that include: dealing with novelty, planning and implementing strategies for performance, monitoring performance, using feedback to adjust future responding, vigilance, and inhibiting task-irrelevant information [12] of lower level, more modular, or automatic functions [14]. Common tasks of daily life require attention, rapid motor planning process, and effective inhibition of irrelevant or inappropriate details. Older adults, however, experience increasing difficulties in maintaining multiple task rules in working memory [15].

These findings imply that in addition to physical forms of training, we should possibly also consider cognitive rehabilitation strategies that aim to influence physical functioning, e.g., walking behavior of older adults [16]. The question remains, however, what the best strategies are, that can support achieving this aim.

Several types of cognitive or cognitive-motor interventions that might be able to improve physical functioning have been proposed in the past: cognitive rehabilitation interventions, training of dual-tasking abilities, and the use of computer games or virtual reality [4,17].

Cognitive rehabilitation, defined by the Brain Injury Interdisciplinary Special Interest Group (BI-ISIG) of the American Congress of Rehabilitation Medicine as a "systematic, functionally-oriented service of therapeutic cognitive activities, based on an assessment and understanding of the person's brain-behavior deficits"[18], has shown to be effective in clinical practice [19,20].

Cognitive rehabilitation interventions have been developed to ameliorate cognitive problems experienced by healthy older adults [21,22], and for adults suffering from traumatic brain injury [19,23,24], with the goal of maximizing their current cognitive functioning and/or reducing the risk of cognitive decline. Some of the cognitive interventions, however, also show transfer effects to physical functioning. Specific motor imagery protocols seem to improve mobility in people with stroke [25].

Cognitive-motor interventions are interventions that combine a cognitive with a physical rehabilitation task, e.g. strength and balance exercises together with cognitive exercises or performing dual-tasking exercises. Interventions that used dual-tasking paradigms demonstrated negative effects on postural control or gait while performing a concurrent cognitive task in older adults [26,27], in patients with brain injury [28,29] and Alzheimer's disease [30]. Several authors have suggested that procedures to improve the dual-task performance of elderly should be included in fall prevention programs [31].

Computerized interventions can be divided into biofeedback based systems or systems that use elements of virtual reality. Becoming aware of various physiological functions by using instruments that provide information on the activity of those same systems is considered biofeedback training. The goal, thereby, is to be able to manipulate these systems at will. Processes that can be controlled include for example dynamic balance on a force platform where visual feedback gives information about the center of pressure movements [32]. In virtual reality, in contrast to biofeedback training, environments are created that allow users to interact with images and virtual objects that appear in the virtual environment in real-time through multiple sensory modalities [32,33]. Playing of computer games induced cognitive benefits in older adults [34], and is proposed as a training strategy that may transfer to physical activity related tasks [4].

All three strategies, cognitive rehabilitation, training of dual-tasking abilities, and computerized interventions, have mainly been applied to individuals with stroke, with traumatic brain injury or elderly. Although it seems intuitive that these groups cannot be compared because of the different underlying causes for their respective brain deficits, this may not actually be the case [35,36]. Studies using a neuropsychological deficit profile methodology suggest that the pattern and extent of cognitive decline associated with these conditions is similar, at least partly, for both cognitive and motor deficits [36,37]. This implies that the treatment approaches needed to remediate the observed deficits are theoretically also comparable.

The objective of this systematic review is to examine the literature regarding the use of cognitive and cognitive-motor interventions to improve physical functioning in older adults and in adults with neurological impairments. The aim is to identify strategies that have the potential to affect physical functioning and that might be used in future intervention type studies for older adults. The specific questions that we asked were: (1) what types of cognitive and cognitive-motor intervention methods have been used to influence physical functioning of older adults or adults with neurological impairments? (2) What is the level of evidence for cognitive and cognitive-motor interventions to influence physical functioning in these populations? (3) What is the methodological quality of these studies?

The underlying assumption that drives these questions is that (changes in) cognition also has an impact on physical functioning.

## Methods

### Data sources and search strategies

In a first step we undertook a scoping review to gain an overview about existing interventions or systematic reviews on this topic. In addition to studies conducted with older adults, interventions with traumatic brain injury patients and patients with stroke were found. Like older adults, people with brain injury or stroke show difficulties with postural balance, exhibit gait insecurities when performing dual-tasks and have cognitive deficits evident in working memory, attention, and information-processing [35,38]. Additionally it has been shown that people with brain injuries show similar characteristics as older adults with an advanced aged-related cognitive decline. The patterns of cognitive decline observed in patients after traumatic brain injury resembles that of classic aging processes [35,39,40]. The search strategy was focused on older adults over the age of sixty-five. Although we are aware that cognitive and physical deficits in patients with brain injury are not fully comparable with the natural aging process we additionally searched further studies with brain injured patients. We also decided to include studies conducted with stroke patients arising from our search, because of their methodological importance for this review and the possible applicability of the applied methods in the general older population.

We developed an individualized electronic search strategy for the Medline/Premedline, PsycINFO, CINAHL, and EMBASE databases in collaboration with a librarian from the Medicinal Library of the University of Zurich. The search was restricted to English, German, and French language literature. There was no limitation of publication date or restriction by study design. The final search was performed in July 2010.

We used medical sub-headings as search terms, including the following main terms for the population: *aged, elder, old, aging, brain/head/craniocerebral injury, trauma*; for cognitive aspects: *cognition, meta-cognition, learning, awareness, attention, self-directed learning, executive function*; for motor functions: *gait, walking, balance, movement, mobility, posture, motor function, accidental falls, training, exercise, physical functioning *and for the interventions of interest: *cognitive therapy/rehabilitation/intervention, problem solving, biofeedback, virtual reality, video game, action game, computerized training, user-computer interface, dual-task *(additional file 1). The search strategy was initially run in Medline/Premedline and then adapted to the search format requirements of the other databases included in this review. The search results were supplemented by articles found through hand search by scanning reference lists of identified studies.

### Study collection

After duplicate citations were removed, two reviewers (GP, EDdB) determined which articles should be included within the systematic review by scanning the titles, abstracts and keywords applying the inclusion and exclusion criteria (table 1). A study was considered eligible for inclusion in the review when it was examining the results of a cognitive or cognitive-motor intervention on physical functioning of older adults. As mentioned in the introduction, we included any study that arose from our search concerning people with traumatic brain injury or stroke patients. Cognitive and cognitive-motor interventions were considered studies that included cognitive rehabilitation or a combination of cognitive rehabilitation and physical exercise, respectively. We did not include studies that solely carried out single tests without an intervention. We adopted the definition of the Brain Injury Interdisciplinary Special Interest Group (BI-ISIG) of the American Congress of Rehabilitation Medicine for cognitive rehabilitation to guide our search. Studies evaluating the effectiveness of pharmacological therapy were excluded. If title, abstract or key words provided insufficient information for a decision on inclusion, the methods section of the full-text article was considered.

**Table 1:** List of inclusion and exclusion details

Area	Inclusion details
Population	Any elderly subjects over 65 years, adult (aged > 18 years) brain trauma patients, studies with stroke patients
Study type	Intervention studies of any type, including case studies and non-randomized trials
Intervention	Cognitive or cognitive-motor rehabilitation intervention (physical exercise must include a cognitive aspect)
Outcomes	Outcomes focus on general physical functioning and mobility of upper or lower extremities

**Exclusion details**	

Purely physical training, interventions without training period (tests), dual-task intervention without concurrent cognitive task, animal studies, reviews, methodological, theoretical or discussion papers, studies that examine the effect of physical exercise on cognition

### Data extraction and data synthesis

The following data were extracted from the studies: (1) characteristics of the studied population: number of participants, disease and age, (2) characteristics of the interventions: the design, frequency and duration of the intervention, co-interventions, and control intervention; (3) characteristics of the outcomes: outcome measures and results (tables 2 and 3). The included studies were divided into three groups: [1] cognitive rehabilitation, [2] dual-task interventions and [3] computerized interventions. Computerized interventions included every study using an electronic game or task that involves interaction with a user interface to generate visual feedback on a display device.

**Table 2:** Included studies reported by design and subject specifications

STUDY	DESIGN	N	SUBJECTS	**AGE **range or mean (years)
Cognitive Rehabilitation Interventions				

**Batson et al 2006**	RCT	6	Community dwelling older adults	65 - 80
**Dunsky et al 2008**	Non-RCT	17	Community dwelling adults with hemiparetic stroke	44 - 79
**Hamel & Lajoie 2005**	RCT	20	Older adults	65 - 90

Dual-task Interventions				

**Shigematsu et al 2008**	RCT	63	Community dwelling older adults	65 - 74
**Shigematsu et al 2008**	RCT	39	Community dwelling healthy adults	65 - 74
**Silsupadol et al 2006**	Case study	3	Older adults with history of falls	82, 90 and 93
**Silsupadol et al 2009**	RCT	21	Older adults	75.0 ± 6.1
**Vaillant et al 2006**	RCT	68	Community dwelling older women with osteoporosis	73.5 ± 1.6
**You et al 2009**	RCT	13	Older adults with history of falls	68.3 ± 6.5

Computerized Interventions				

**Bisson et al 2007**	Pre-Post	24	Community dwelling older adults	VR: 74.4 ± 3.65; BF: 74.4 ± 4.92
**Broeren et al 2008**	Pre-Post	22	Community dwelling adults with stroke	67.0 ± 12.5
**Buccello-Stout et al 2008**	RCT	16	Older adults	66 - 81
**Clark et al 2009**	Case study	1	Woman resident of a nursing home with unspecified balance disorders	89
**de Bruin et al 2010**	Two groups control	35	Older adults living in a residential care facility	IG: 85.2 ± 5.5; CG: 86.8 ± 8.1
**Deutsch et al 2009**	Case study	2	Chronic phase post-stroke	34 and 48
**Hatzitaki et al 2009**	RCT	48	Community-dwelling healthy older women	70.9 ± 5.7
**Hinman 2002**	RCT	88	Community-dwelling older adults	63 - 87
**Jang et al 2005**	RCT	10	Patients with stroke	57.1 ± 4.5
**Kerdoncuff et al 2004**	RCT	25	Patients with stroke	59.5 ± 13.5
**Lajoie 2003**	RCT	24	Community-dwelling elderly	IG: 70.3; CG: 71.4
**Mumford et al 2010**	Case study	3	Patients with TBI	20, 20 and 21
**Sackley et al 1997**	RCT	26	Patients with stroke	41-85
**Srivastava et al 2009**	Pre-Post	45	Patients with stroke	45.5 ± 11.2
**Sugarman et al 2009**	Case study	1	Patent with stroke	86
**Talassi et al 2007**	Case-control	54	Community-dwelling older adults with MCI or MD	42 - 91
**Wolf et al 1997**	RCT	72	Independently living older adults	CBT: 77.7 ± 6.5; TC: 77.7 ± 5.6; CG: 75.2 ± 4.9
**Yang et al 2008**	RCT	20	Patients with stroke	30 - 74
**Yong Joo et al 2010**	Pre-Post	16	Rehabilitation inpatients within 3 months post-stroke	64.5 ± 9.6

**Abbreviations**: RCT = Randomized Controlled Trial; Non-RCT = Nonrandomized Controlled Trial; TC = Tai Chi; VR = Virtual reality; BF = Biofeedback; IG = Intervention Group; CG = Control Group

**Table 3:** Included studies reported by subjects, outcome measures, intervention, control and results

STUDY	SUBJECTS	OUTCOME MEASURES	INTERVENTION	CONTROL	RESULTS
**Cognitive Rehabilitation Interventions**					

**Batson et al, 2006 **[44]	- n = 6; community-dwelling elderly - age range: 65-80 years	- Standardized measures of balance, gait speed and balance confidence - BBS, ABC - TUG	Mental imagery plus physical practice; 6 weeks: 2x/week for 50 min	Health education plus physical practice 6 weeks: 2x/week for 50 min	- Significant results for TUG only for the group as a whole - No significant results for either group or for the group as a whole for remaining measures

**Dunsky et al, 2008 **[50]	- n = 17; community-dwelling adults with hemiparetic stroke - age range: 44-79 years	- Spatiotemporal and kinematic gait parameters - Tinetti POMA - FMA - Modified FWCI	Motor imagery training; 6 weeks: 3 x/week for 20 min	None	- Spatiotemporal parameters: significant improvements in mean gait speed at baseline and follow-up; stride length, paretic and non-paretic step length increased significantly at post-intervention - Significant increase of sagittal ROM of the paretic knee joint - Significant increase of gait symmetry after intervention - Treatment effect size was moderate for most of the variables

**Hamel & Lajoie, 2005 **[51]	- n = 20; older adults - age range: 65-90 years	- A/P & M/L postural oscillations - Reaction time to auditory stimuli - BBS - ABC	Mental imagery training; 6 weeks: daily practice	No involvement in any type of training	- MI-group became more stable after training, while sway of control group increased when compared to pre-test. - A/P postural oscillation significantly decreased in MI-group - Significant decrease in reaction time task for MI-group - No significant outcomes on BBS and ABC scales

**Dual-task Interventions**					

**Shigematsu et al, 2008 **[58]	- n = 63; community dwelling older adults - age range: 65-74 years	- Physical tests of balance, leg strength and coordination - Self-reported occurrence of falls or trips - Step-recording with pedometers	Square-Stepping Exercise (SSE); 12 weeks: 2x/week for 70 min	Supervised walking (W); 12 week: 1x week for 70 min	- Functional fitness of lower extremities improved more in SSE than in W - No significantly lower rate of falls per trip for SSE compared to W.

**Shigematsu et al, 2008 **[59]	- n = 39; community-dwelling healthy adults - age range: 65-74 years	- Chair stands, Leg extension power, Single-leg balance with eyes closed, functional reach, standing up from a lying position, stepping with both feet, walking around two cones, 10 m-walk, Sit&Reach	Square-Stepping Exercise (SSE); 12 weeks: 2x/week for 70 min	Strength and balance training; 12 weeks: 2x/week for 70 min	- SSE: significant within-group improvement in one-leg balance - SB: Significant improvement of functional reach - Performances on remaining test were significantly better for both groups.

**Silsupadol et al, 2006 **[62]	- n = 3 older adults with self-reported history of falls or concerns about impaired balance - age: 82, 90 and 93 years	- Mediolateral COM displacement und single-task (ST) and dual-task (DT) - BBS, ABC - DGI - TUG	Dual-task balance training with fixed- (FP) or variable-priority (VP); 4 weeks: 3x/week for 45 min	Single-task balance training; 4 weeks: 3x/week for 45 min	- Balance improved in all 3 participants, BBS, DGI and ABC scores increased - Time to complete TUG decreased under both conditions (participants who received DT-Training showed more improvement in TUG under DT than under ST and vice versa) - Subject who received DT-training using VP, showed improvements on other dual tasks that were not directly trained (novel task) - Follow-up (2 weeks): time to perform TUG decreased for all subjects - Follow-up (3 months): Clinical measures of balance were retained; TUG in subject with FP further improved (9%)

**Silsupadol et al, 2009a&b **[60,61]	- n = 21; elderly adults - mean age: 75 ± 6.1 years	- Self-selected gait speed under single and dual task conditions - Gait temporal-distance measurements - BBS, ABC - Average angle of frontal plane COM position and ankle joint center (AJC)	Dual-task balance training with fixed- (FP) or variable-priority (VP); 4 weeks: 3x/week for 45 min	Single-task balance training; 4 weeks: 3x/week for 45 min	- All participants improved gait speed under ST conditions. - DT-groups walked significantly faster under DT conditions. No significant difference in gait speed under DT conditions for ST-group - All participants improved balance under ST-conditions - ABC Scale: ST group increased their level of confidence more than DT groups - BBS Scale: improvements in BBS were comparable across training groups - Follow-up: DT-training with VP instructions demonstrated a training effect on DT-gait speed at the end of the second week of training and also after 3 months follow-up - All groups showed a significantly smaller AJC-angle after training when walking under ST conditions - Under DT-conditions reduction of AJC-angle was significant for all groups, but was greater for the VP-group than for the ST-group and FP-group - No significant effects on AJC-angle in a novel (untrained) DT-condition for all groups.

**Vaillant et al, 2006 **[66]	- n = 68; community-dwelling older women with osteoporosis - mean age: 73.5 ± 1.6 years	- TUG & TUG-DT - One Leg Balance (OLB) and OLB with concurrent task (OLB-DT)	Physical exercise while counting, memorizing or reciting (dual task); 6 weeks: 2x/week	Physical exercises (single task); 6 weeks: 2x/week	- Adding cognitive tasks did not significantly alter the effects of the exercise program - 2 weeks follow-up: Significant improvements for all outcome measures in both groups; TUG time improved more in single-task group than in dual-task group - 3 months follow-up: Improvements in TUG-DT significantly greater in dual-task group than in the single-task group

**You et al, 2009 **[70]	- n = 13; older adults with history of falls - mean age: 68.3 ± 6.5 years	- Gait speed - AP-/ML-COP deviation	Cognitive Gait Intervention (CGI); 6 weeks: 5x/week for 30 min	Placebo version of CGI; 6 weeks: 5x/week for 30 min	- No significant difference in the ML-COP or AP-COP deviation measures neither in control nor experiment group; - Significant increase in gait speed in control group but not in experimental group

**Computerized Interventions**					

**Bisson et al, 2007 **[32]	- n = 24; community dwelling older adults - mean age: VR 74.4 ± 3.65 years, BF 74.4 ± 4.92 years	- Static balance - Simple auditory reaction time task - CB&M	Dynamic balance training with visual biofeedback (BF) or in virtual reality (VR); 10 weeks: 2x/week for 30 min	None	- Mean CB&M scores for both groups increased significantly from baseline to post-training and retention, no difference between groups - Static balance: no differences between groups and no training effect on variability of COP displacement; Significant task effect and interaction between directions of sway and tasks - Reaction time: no group effect; significant main effect of time; reaction time at baseline significantly higher compared to post-training and retention; both groups improved their reaction time equally

**Broeren et al, 2008 **[45]	- n = 22; community dwelling persons with stroke - mean age: 67 ± 12.5 years	- Manual Ability measurements (BBT and ABILHAND) - Trail Making Test B - Kinematics of upper extremities (velocity, hand-path ratio etc.)	3D computer game play with haptic device and unsupported upper extremities; 4 weeks: 3 x/week for 45 min	Continued participation in usual physical activities	- BBT: Increase in treatment group by 9% - ABILHAND: No significant changes in both groups - TMT-B: median time decreased for completing the task in both groups - Kinematics: Time to complete the VR task and HPR decreased significantly in treatment group - Hand trajectories are qualitatively more restrained, self-controlled, smoother and less clutters after training

**Buccello-Stout et al, 2008 **[46]	- n = 16; older adults - age range: 66 - 81 years	- Time to complete an obstacle course with 13 soft obstacles - Number of penalties on obstacle course	Walking straight on a treadmill in a rotating virtual room; 4 weeks: 2 x/week for 20 min	Walking straight on a treadmill in a static virtual room; 4 weeks: 2 x/week for 20 min	- Average time scores to complete obstacle course and average penalty scores significantly decreased in experimental group after intervention and at retention (4 weeks)

**Clark et al, 2009 **[47]	- n = 1; woman resident of a nursing home with unspecified balance disorders - age:89 years	- BBS, ABC - DGI - TUG - MMSE	Nintendo Wii Bowling game; 2 weeks: 3x/week for 60 min	None	- Improvements in all outcome measures - Self-reported improvements in balance, ambulation ability and confidence

**de Bruin et al, 2010 **[48]	- n = 35; older adults living in a residential care facility; - mean age: CGD 85.2 ± 5.5 years, UC 86.8 ± 8.1 years	- Gait temporal-distance measurements - Dual task costs of walking - ETGUG - FES-I	Computer game dancing (CGD) plus progressive resistance training; 12 weeks: 2x/week for 45-60 min	Usual care physical intervention (UC); 12 weeks: 1x/week for 30-45 min	- DTC: Significant decrease in DTC of walking velocity and stride time in CGD-group. No significant changes in DTC of cadence and step time in both groups. - ETGUG: no significant time effect in both groups - FES-I: no significant time effect in both groups

**Deutsch et al, 2009 **[49]	- n = 2; in chronic phase post-stroke patients - age: 48 and 34 years	- Gait speed - Six-minute walk test (meters) - BBS, ABC - DGI - TUG and TGU-DT	Nintendo Wii Sports and Wii Fit Programs; 4 weeks: 3x/week for 60 min	Balance and coordination activities in different conditions; 4 weeks: 3x/week for 60 min	- Gait speed increased for both participants (retained at follow-up) - Gait endurance increased modestly for both participants - DGI and ABC scores increased for both participants - TUG and TUG-DT time decreased for both participants; Control subject showed further improvement at post-test

**Hatzitaki et al, 2009 **[52]	- n = 48; community-dwelling healthy older women - mean age: 70.89 ± 5.67 years	- Static postural sway data: COP displacement in A/P and M/L direction - Angular excursion of lower leg, pelvis and trunk	Balance training on platform with visual feedback in A/P or M/L direction; 4 weeks: 3x/week for 25 min	No involvement in any type of training	- Normal quiet stance: No significant changes in COP displacement and angular kinematics in either of the two training groups. - Significant effect of training on interlimb COP asymmetry in A/P-group - Sharpened Romberg Stance: Significant reduction of COP displacement in A/P-group, no adaptations in M/L-group. A/P group showed significantly decreased peak amplitude and SD of lower leg rotation in the pitch direction and of trunk's mediolateral rotation. No significant changes in the M/L-group

**Hinman, 2002 **[53]	- n = 88; community-dwelling elderly - age range: 63-87 years	- BBS - MFES - Timed 50-foot walk test (TWT) - Simple reaction time	Computerized Balance Training (CBT) or Home program of balance exercises (HEP); 4 weeks: 3 x/week for 20 min	No involvement in any type of training	- Subjects in both training groups showed slight improvements in all measures. Subjects of control group improved to a lesser degree.

**Jang et al, 2005 **[54]	- n = 10; patients with hemiparetic stroke - mean age: 57.1 ± 4.5 years	- BBT - FMA - Manual Function Test - Several fMRI data	VR game exercise with IREX system focusing on reaching, lifting and grasping; 4 weeks: 5x/week for 60 min	No involvement in any type of training	- Significant difference between the groups, VR-group improved in motor functions, control group did not show any change - Cortical activation was reorganized from contralesional to ipsilesional activation in the laterality index

**Kerdoncuff et al 2004 **[71]	- n = 25; patients with stroke - mean age: 59.5 ± 13.5	- FMA - Gait evaluation - Barthel Index - Measurement of functional independence (MFI) - Sway measurements on force platform	Progressive balance training with visual biofeedback plus traditional training; 3 weeks: 5x/week	Traditional training; 3 weeks: 5x/week	- Improvements in gait speed for control group, decrease for intervention group - Improvements in FMA, MFI and Barthel Index for both groups - Improvements of force platform parameters with closed eyes

**Lajoie, 2003 **[55]	- n = 24; community-dwelling elderly - mean age: IG 70.3 years, CG 71.4 years	- BBS, ABC - Auditory-verbal reaction test - Postural sway data	Computerized Balance Training; 8 weeks: 2x/week for 60 min	No involvement in any type of training	- BBS: Significant difference for CBT-group after intervention - ABC: No significant changes - Significant decrease of reaction time in CBT-group after intervention - Postural sway: No significant changes in both groups

**Mumford et al, 2010 **[56]	- n = 3; patients with TBI - mean age: 20.3 years	- Movement accuracy - Movement speed - Movement efficiency - BBT - MAND	Table-top VR-System for moving objects to cued locations with augmented movement feedback; 12 weeks: 1x/week for 60 min	None	- Accuracy: Improvements after intervention and maintained in 2 of 3 patients - Speed: No improvement after intervention for either hand - Efficiency: Improved performance efficiency for all participants after intervention - BBT: moderate improvements - MAND: moderate improvements

**Sackley et al, 1997 **[57]	- n = 26; stroke patients - age range: 41-85 years	- Stance symmetry and sway - Rivermead Motor Assessment - Nottingham 10 Point ADL Scale	Balance training using visual feedback; 4 weeks: 3x/week for 60 min	Balance training without visual feedback; 4 weeks: 3x/week for 60 min	- Treatment group demonstrated significantly better performance when compared with controls for stance symmetry and for functional performance (ADL and Gross Function scores) - Sway values showed a tendency to greater improvement

**Srivastava et al, 2009 **[63]	- n = 45; stroke patients - mean age: 45.51 ± 11.24 years	- BBS - Balance Index - Dynamic Limits of Stability scores - Walking ability - Barthel Index	Balance training on force platform with visual feedback; 4 weeks: 5x/week for 20 min	None	- Statistically significant differences at the end of training for all outcome measures - Statistically significant differences for all outcomes at 3 months follow-up

**Sugarman et al, 2009 **[64]	- n = 1; woman 5 weeks after stroke - age: 86 years	- BBS - Functional Reach - TUG - Postural Stability Index (STI) - Stability Score (ST)	Nintendo Wii Fit balance training plus standard physical therapy with emphasis on functional activities; 4 × 45 min	None	- Modest improvements in BBS and Functional Reach tests - TUG time decrease - Modest improvements in postural stability tests

**Talassi et al, 2007 **[65]	- n = 54; community-dwelling older adults with mild cognitive impairment (MCI) or mild dementia (MD) - age range: 42-91 years	- PPT - Basic and instrumental ADL	Computerized cognitive training (CCT), occupational therapy (OT) and behavioral training (BT); 3 weeks: 4x/week for 30-45 min	same program with physical rehabilitation program (PT) instead of CCT	- Participants with MCI showed significant improvements in PPT - Unspecific control program showed no significant effects

**Wolf et al, 1997 **[67]	- n = 72; independently living older adults; - mean age: CBT 77.7 ± 6.5 years, TC 77.7 ± 5.6 years, Control Group 75.2 ± 4.9 years	- Postural stability measurements under defined conditions - Fear of Falling Questionnaire	Computerized Balance Training (CBT) or Tai Chi (TC); 15 weeks: CBT 1x/week for 60 min, TC 2x/week for 60 min	Educational intervention (ED); 15 weeks: 1x/week for 60 min	- CBT: improved postural stability - TC: no improvements in postural stability, but reduction of fear of falling occurred

**Yang et al, 2008 **[68]	- n = 20; adults with stroke - age range: 30-74 years	- Walking speed - Community walk test (CWT) - Walking Ability Questionnaire (WAQ) - ABC	Virtual reality-based treadmill training; 3 weeks: 3x/week for 20 min	Treadmill training; 3 weeks: 3x/week for 20 min	- VR-Group: significant improvement in all outcomes post-training and significant improvements in walking speed, CWT and WAQ score 1 month after completion of program - CG: significant improvements in CWT post-training and in follow-up period, significant improvements of WAQ score at follow-up

**Yong Joo et al, 2010 **[69]	- n = 16; rehabilitation inpatients within 3 months post-stroke - mean age: 64.5 ± 9.6 years	- FMA - Motricity Index - Modified Ashworth Scale (MAS) - Visual Analogue Scale for upper limb pain	Upper limb exercises with Nintendo Wii in addition to usual rehabilitation; 2 weeks: 6x/week for 30 min	None	- Significant improvements in the FMA and Motricity Index scores

**Abbreviations**: BBS = Berg Balance Scale; ABC = Activities-specific Balance Confidence Scale; CB&M = Functional Balance and Mobility; COP = Centre of Pressure; COM = Centre of Mass; DGI = Dynamic Gait Index; TUG = Timed Up and Go Test; TUG-DT = Timed Up and Go Test Dual Task; ETGUG = Expanded Timed Up and Go Test; MMSE = Mini Mental State Examination; ADL = Activities of Daily Living; BBT = Box and Block Test; MAND = Mc Carron Assessment of Neuromuscular Dysfunction; FMA = Fugl-Meyer Assessment of Upper Limb Motor Function; FES-I = Falls Efficacy Scale International; MFES = Tinetti's Modified Falls Efficacy Scale; POMA = Performance Oriented Mobility Assessment; FCWI = Functional Walking Categories Index; PPT = Physical Performance Test

Because we expected the interventions and reported outcome measures to be markedly varied, we focused on a description of the studies and their results, and on qualitative synthesis rather than meta-analysis.

### Assessment of study quality

As the basis for our critical appraisal of the studies, a checklist designed for assessing the methodological quality of both randomized and non-randomized studies of healthcare interventions developed by Downs and Black [41] was used. The checklist assesses biases related to reporting, external validity, internal validity, and power. Seven items concerning follow-up analyses (items 9, 17 and 26), allocation concealment (items 14 and 24), adverse effects (item 8), and representativeness of treatment places and facilities (item 13) were not considered in this review. The items were excluded because we were not primarily interested in possible long-term effects of cognitive or cognitive-motor interventions but rather in short-term effects of the interventions on motor functioning. The blinding of participants and investigators, the assessment of adverse effects, and the representativeness of the treatment places were also excluded. We considered these as being of minor significance for this review.

The remaining 20 items were applied by two reviewers (GP/EDdB) to assess the methodological quality of the studies (additional file 2). The total possible score was 22 points. The scoring for statistical power (item 27) was simplified to a choice between 0, 1 or 2 points depending on the level of power to detect a clinically important effect. The scale ranged from insufficient (β < 70% = 0 points), sufficient (β = 70-80% = 1 point) or excellent (β > 80% = 2 points). To assess the level of agreement between the investigators a Cohen's kappa analysis was performed on all items of the checklist. In accordance with Landis and Koch's benchmarks for assessing the agreement between raters a kappa-score of 0.81 - 1.0 was considered almost perfect, 0.61 - 0.8 was substantial, 0.41 - 0.6 was moderate, 0.21 - 0.4 was fair, 0.0 - 0.2 slight and scores <0 poor [42]. Disagreements were resolved by consensus.

The PRISMA-statement was followed for reporting items of this systematic review [43].

## Results

### Study selection

**Figure 1:**
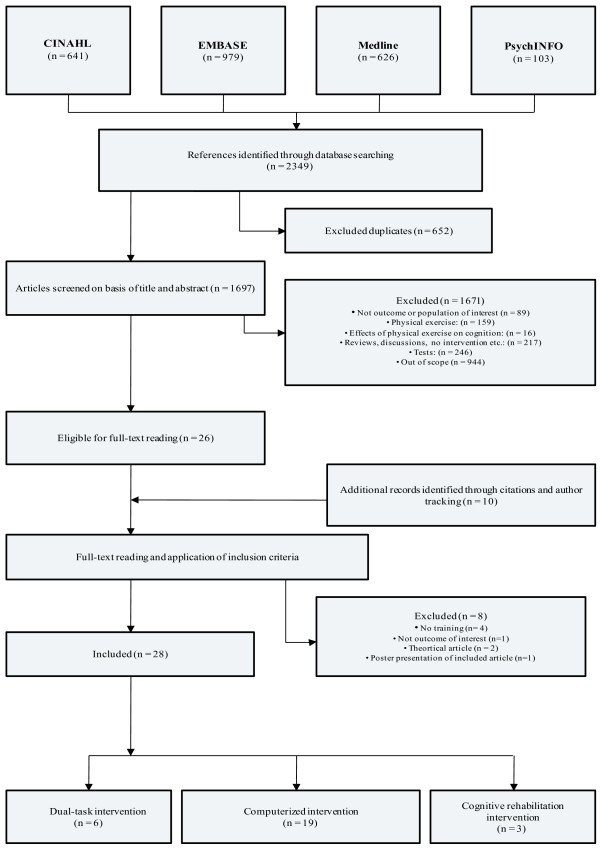
Study selection flow chart

The search provided a total of 2349 references (figure 1). After adjusting for duplicates, 1697 remained. Of these 1671 were discarded because they provided only physical exercise (n = 159), did not discuss outcomes or population of interest (n = 89), constituted review articles or were no interventional studies (n = 217), executed only single tests (n = 246) or were clearly out of scope of this review (n = 944). The remaining 26 potentially relevant articles were supplemented by 10 additional references retrieved by citations and author tracking, resulting in a total of 36 articles being eligible for full-text reading. After full-text reading eight articles were excluded because they did not report outcomes of interest (n = 1), applied no intervention (n = 1), applied no training (n = 4), or were theoretical articles (n = 2). One article appeared to be a written summary of a poster presentation and represented an included article (n = 1).

### Characteristics of included studies

Of the 28 studies finally selected for the review 27 were published in English [32,44-70] and one in French [71]. The publication dates range from 1997 [67] to 2010 [48,56,69]. In the selected studies participants were older adults partially with history of falls [47,70], balance disorders [47,62], with mild cognitive impairments [65] or osteoporosis [66]. Ten studies were concerned with patients after stroke [45,49,50,54,57,63,64,68,69,71] and one study with traumatic brain injury patients [56].

From the 28 included articles three used an isolated cognitive rehabilitation intervention [44,50,51], seven articles used a dual-task intervention [58-62,66,70] and 19 applied a computerized intervention [32,45-49,52-57,63-65,67-69,71]. From the seven articles concerning dual-tasking two articles arise from the same intervention [60,61] leading us to regard it as one single study.

In 22 studies, a cognitive rehabilitation intervention, dual-task training or a computerized intervention were used as the only intervention for the participants [32,45-47,49-52,54-63,66-70]. In six studies the interventions were applied as additional items to a traditional physical or balance training [44,48,53,64,65,71]. The reported outcomes involved different assessments of balance, gait or functional mobility. Balance was assessed with the help of postural sway measurements [32,44,51,52,57,62,67,71], with the Berg Balance Scale [44,47,49,51,53,55,60-64], with the Activities-specific Balance Confidence Scale [44,47,49,51,55,60-62,68], with the Functional Balance and Mobility test [32], with the Balance Index [63] and with one-leg-stance tests [59,66]. Gait measurements included measurements of kinematic parameters [44,48-50,60,61,68,70], the Timed Up & Go Test [44,47-49,62,64,66], the Dynamic Gait Index [47,49,62], or step-recording with pedometers [58]. Functional Mobility assessments were determined by manual ability measurements [45,54], functional reach tests [64], the Physical Performance Test [65], the Rivermead Motor Assessment [57], The Nottingham 10 Point ADL Scale [57], the Box and Block Test [45,54,56] and the Fugl-Meyer Assessment of Upper Limb Motor Function [50,54,69,71].

### Methods used and their effects

#### A. Cognitive rehabilitation interventions

From the three articles evaluating the effects of a cognitive rehabilitation intervention on motor outcomes, two examined the effects of mental imagery on physical functioning of older adults aged between 65 and 90 years [44,51]. In the third study, the participants were community-dwelling adults between 44 and 79 years of age suffering from hemiparetic stroke [50]. The three studies investigated the effect of mental imagery training on postural balance [44,51] and on gait [44,50]. Mental imagery training consisted of either visual imagery training, i.e. participants are expected to view themselves from the perspective of an external observer, or of kinesthetic imagery exercise, i.e. participants imagine experiencing bodily sensations that might be expected in the exercise. The trainings lasted six weeks with a training frequency ranging from daily [51], twice weekly [44] to three times weekly [50]. Two studies used a pure cognitive rehabilitation method [50,51] whereas one study combined mental practice with additional physical exercise [44].

The studies show reduction of postural sway [51], and improvements in gait speed [44] and gait symmetry [50]. No improvements were shown for balance confidence [44].

Hamel and Lajoies' [51] results show a significant reduction of antero-posterior postural oscillations suggesting that mental imagery training over a six-week period helps to improve postural control of the elderly. The study of Batson et al. [44] combined mental imagery with physical exercise. The control group underwent a health education program in addition to the physical training. Gait speed, expressed by improvement in Timed Up-and-Go test performance, increased for all study participants. These results imply that the improvement in gait speed were attaint through the physical practice regardless of whether combined with mental imagery or not. This conjecture is supported by the fact that the two groups under observation converge to each other for the Timed Up-and-Go test measures following the intervention. In the pretest phase, there was a large, meaningful difference for the Timed Up-and-Go test between the mental imagery and physical practice subjects (Cohen's *d *= 1.2) that decreases to Cohen's *d *= 0.55 at the end of intervention. The results showed no improvement in balance confidence, as expressed by non significant results neither on the Berg Balance Scale nor on the Activities-specific Balance Confidence Scale. The study of Dunsky et al. [50] showed improvements of spatiotemporal gait parameters and gait symmetry in people with chronic poststroke hemiparesis after mental imagery. There was no control group in this study to support these results.

#### B. Dual-task interventions

The methods varied from walking or balancing with a concurrent mental task like memorizing words, reciting poems, or computing mental arithmetic tasks [60-62,66,70] to a square-stepping exercise where participants executed forward, backward, lateral and oblique step patterns on a thin felt mat [58,59]. The training lasted between 4 weeks [60-62], 6 weeks [70] or 12 weeks [58,59,66]. No dual-task study was found on stroke patients or people with traumatic brain injury. The study of Shigematsu et al. [58] showed improvements in functional fitness of lower extremities. The results on gait patterns and postural sway are controversial. Silsupadol et al. [60-62] showed improvement of gait speed under dual-task conditions and a reduction of body sway, whereas You et al. [70] and Vaillant et al. [66] found no improvements in gait and stability after a dual-task intervention. No other physical outcomes were reported.

The studies conducted by Silsupadol et al. [60-62] compared three different balance training approaches: single-task balance training, dual-task balance training with fixed-priorities and dual-task balance training with variable-priority. Single-task training consisted of exercises for body stability with or without object manipulation and/or body transport. In the dual-task condition, concurrent auditory and visual discrimination tasks and computing tasks were added to the balance training. In the fixed-priority condition the subject was instructed to direct the attention with equal priority to both the postural and additional tasks. In the variable-priority condition half the training was done with the instruction to mainly prioritize the postural task and the other half with the instruction to mainly prioritize the additional task. All participants improved self-selected gait speed under single-task testing conditions. Under dual-task testing conditions, however, only participants who received dual-task training showed significant improvements in self-selected gait speed (with moderate effect sizes of 0.57 between single-task and fixed-priority and 0.46 between single-task and variable-priority). All groups significantly improved on the Berg Balance Scale under single-task conditions. Participants in the variable-priority training group additionally showed an average of 56% reduction in body sway compared to only 30% of the fixed-priority and single-task group. Overall, the study showed that variable-priority instruction was more effective in improving both balance and physical performance under dual-task conditions than either the single-task or the fixed-priority training approaches. In contrast to the fixed-priority training group, the variable-priority group showed long-term maintenance effects on dual-task gait speed for three months after the end of training.

In contrast to the results of Silsupadol et al., You and colleagues [70] found no improvements in gait and stability after their dual-task intervention that lasted six weeks. Results of the gait tests showed a significant increase in gait velocity in the control group which underwent single-task training but not in the experimental group. No statistically significant differences in the deviation of mediolateral and anteroposterior centre of pressure were found between the groups. Vaillant et al. [66] did not find additional improvements through the addition of a cognitive task to the physical task either. The exercise sessions were effective in improving performance on two balance tests, improvements, however, were not attributable to the dual-task training.

Shigematsu et al. [58,59] developed an alternative approach to exercise for dual-task abilities in community-dwelling older adults. A square stepping exercise was performed on a thin mat with the instruction to step from one end of the mat to the other according to a step pattern provided, which could be made progressively more complex. Results showed that square stepping exercise was equally effective as strength training to improve lower-extremity functional fitness. Compared to a weekly walking session, however, participants of the square stepping exercise group showed a greater improvement in functional fitness of the lower-extremity.

#### C. Computerized interventions

Nineteen studies investigated the effects of a computerized intervention to improve physical abilities. The studies were distributed over the populations of interest as follows: nine interventions treating older adults [32,46-48,52,53,55,65,67], nine interventions treating patients with stroke [45,49,54,57,63,64,68,69,71] and one study treating young adults with traumatic brain injury [56]. Fifteen studies investigated the effects on lower extremities [32,46-49,52,53,55,57,63-65,67,68,71], whereas four studies analyzed the effects on upper extremities [45,54,56,69]. The interventions included various methods and ideas for the implementation of computers into a training session. Talassi et al. [65] used a computerized cognitive program [72,73], to stimulate cognitive functions, e.g. visual search, episodic memory or semantic verbal fluency, by a specific group of exercise for older adults with mild cognitive impairments or mild dementia. Buccello-Stout et al. [46] used a sensorimotor adaptation training to improve functional mobility in older adults. Participants walked on a treadmill while viewing a rotating virtual scene providing a perceptual-motor mismatch [46].

Seven studies used the method of computerized dynamic balance training with visual feedback technique [52,53,55,57,63,67,71]. The tasks required to move through weight-shifting a cursor on a screen representing the centre of pressure (COP) position to specified targets [32,53,55,63,67] or on a predefined sine wave trajectory [52]. In one study, the feedback signal displayed the weight distribution and weight shifting with moving columns, showing stance symmetry [57]. In another study researchers designed the task of visual feedback training in a more playful way, projecting the cursor for centre of pressure as a caterpillar moving on the screen [71]

A total of ten studies described an approach which included interactive virtual reality games or applications [32,45,47-49,54,56,64,68,69]. Seven studies out of this ten were conducted on stroke patients [32,45,49,54,64,68,69], two studies on older adults [47,48] and one on patients with traumatic brain injury [56]. The virtual reality applications were varied. There were elaborated and expensive systems, enabling the participants to see themselves in the virtual environment and to play games like juggling a virtual ball [32] or saving a ball as a soccer keeper [54]. Virtual devices consisting of a semi-immersive workbench with which participants were able to reach and interact with three-dimensional objects [45], a table-top virtual-reality based system requiring the patients to move an object to cued locations while receiving augmented movement feedback [56] and virtual-reality based treadmill training [68]. Furthermore, commercially available low-cost interactive video game console systems [47,49,64,69] or dance simulation games [48] were applied.

The computerized cognitive training program proposed by Talassi et al. [65] produced an improvement in functional status, measured by the Physical Performance Test [74], in patients with mild cognitive impairments, while a physical rehabilitation program did not show any significant effects. The sensorimotor adaptation training for older adults developed by Buccello-Stout et al. [46] resulted in better performance on an obstacle course after the intervention compared to the control group, who walked on the treadmill without rotation of the virtual scenario.

Some of the interventions providing balance training with visual feedback improved simple auditory reaction time [32,55] postural balance and stability [32,55,57,63,67,71], gait speed [63], functional status, and performance [55,57,63,71]. The intervention conducted by Hatzitaki et al. [52] revealed that weight-shifting training in antero/posterior direction only induces improvements in standing balance of older adults. In contrast, the studies of Lajoie et al. [55] and Bisson et al. [32] showed no improvements in postural sway after computerized balance training in older adults. Hinman and colleagues [53] also found no improvements neither in balance, gait speed nor in simple reaction time compared to the control group. The results of Kerdoncuff et al. [71] even showed a reduction of gait speed in stroke patients treated with visual biofeedback compared to an increase in gait speed for the control group treated with a traditional physical rehabilitation program.

The methods using immersive computer technologies resulted in improved motor functions of upper extremities and a cortical activation by the affected movements from contralesional to ipsilesional activation in the laterality index after virtual reality intervention in patients with chronic stroke [54]. Older adults benefited from training in terms of improved functional abilities, postural control and simple auditory reaction times [32]. A virtual rehabilitation program with the help of a semi-immersive virtual reality workbench, in a non-hospital environment, resulted in qualitatively improved manual trajectories and increased movement velocity of the trained upper extremities for patients with stroke, without any transfer to real-life activities [45].

A virtual reality-based treadmill intervention conducted by Yang et al. [68] requested patients with stroke to walk on a treadmill while observing a virtual scenario of the typical regional community. The scenarios consisted of lane walking, street crossing, striding across obstacles, and park stroll with increasing levels of complexity. Participants improved their walking speed and walking ability at post-training as well as after one month after the training.

Effects on motor functions were also observed in studies using so-called off the shelf computer game systems. Four studies proposed a training program using the Nintendo Wii console [47,49,64,69]. Three of them were case studies and exemplified that a training with a commercially available computer game system can be applied for older adults [47] and for the treatment of balance problems after stroke [49,64]. The participants performed physical training using the Wii Fit system. Using the approach of the weight-shifting method with visual feedback, the Wii Fit games were controlled by shifting body weight on the platform combined with a challenging game [64]. The activities on the Nintendo Wii console were selected to practice balance, coordination, strengthening, endurance or bilateral upper extremity coordination [47,49]. Subjects very much enjoyed the interventions resulting in better balance and mobility performance [47,64], improvements in gait speed, gait endurance and balance [49]. A recently published study using the Nintendo Wii console [69] resulted in improvements in upper extremity functions in post stroke patients.

A study conducted by de Bruin et al. [48] studied the transfer effects on gait characteristics of elderly who executed a traditional progressive physical balance and resistance training with integrated computer game dancing. The task of the dancing game consisted of stepping on arrows on a dance pad. Results indicated a positive effect of the computer game dancing training on relative dual-task costs of walking, e.g., stride time and step length. The more traditional physical training showed no transfer effects on dual-task costs related gait characteristics.

### Quality evaluation

The agreement on study quality between the two reviewers was almost perfect. The estimated Kappa value was 0.96 with a confidence interval ranging between 0.95 and 0.98. The percentage of agreement between the two reviewers was 98.18%. The quality scores ranged from 7 to 22 points out of a maximum of 22. The mean quality score was 13.46 points (range: 7-22 points), the median value was 6.5 points and the mode was 12 points. The mean score for reporting was 6.57 points (maximum: 9 points; range: 4-9 points), for external validity 0.68 (maximum: 2 points; range: 0-2 points), for internal validity (bias) 3.71 points (maximum: 5 points; range: 2-5 points), for internal validity (confounding) 2.25 (maximum: 4 points; range: 0-4 points).

Additional file 2 summarizes the results of the quality assessment for the three intervention types: cognitive rehabilitation interventions, dual-task interventions, and computerized interventions.

## Discussion

An increased incidence of falls among older adults is one of the most serious problems of mobility impairment. It has been suggested that effective programs to prevent falls in older adults should focus on training both physical and cognitive aspects. The aim of this systematic review was to examine the literature on the effects of cognitive and motor-cognitive interventions to improve physical functioning of older adults with additional insights from studies conducted with brain injured adults or patients with stroke.

Our search resulted in relatively few studies that evaluated a cognitive or a motor-cognitive intervention. Twenty-eight articles were found including studies with older adults or patients with neurological impairments. Our results show that the method of combining physical exercise with cognitive elements to improve physical functioning is not yet systematically part of the current interventions for older adults or patients with neurological impairments. The methodological heterogeneity and the numerous feasibility studies are indicators for a topic still being in its fledgling stage.

The results of the few studies identified in this review, however, justify larger studies with older adults. There is evidence that cognitive or motor-cognitive interventions positively affect physical functioning, such as postural control, walking abilities and general functions of upper and lower extremities. The majority of the included studies resulted in improvements of the assessed functional outcome measures. The next sections will discuss the three different intervention types applied in more detail.

### Cognitive rehabilitation interventions

The prevalent technique used was mental imagery, which involved the participants imagining themselves in a specific environment or performing a specific activity, without actually performing it [75]. Brain-imaging studies showed that comparable brain areas are activated during actual performance and during mental rehearsal of the same tasks [76,77]. Hamel and Lajoie's [51] suggest that after mental imagery the motor control task becomes more automatic, leading to a decrease in attentional demands directed toward the control of the motor task.

Our search resulted in three relevant studies that applied mental imagery. In one study [44] improvements in physical functioning were shown in both the intervention and the control group and in a second study [50], a missing control group made it impossible to assess whether improvements in physical functioning were attained through mental practice or not. Thus, as for now it is not possible to determine whether an isolated cognitive rehabilitation intervention based on mental imagery is able to improve physical functioning in older adults. There is evidence about the effectiveness of mental imagery in improving physical functioning of other populations than older adults [25,75]. It seems fair to state that larger randomized control studies should be performed in order to provide more insights in the impact of mental imagery in older adults.

### Dual-task interventions

Research has shown that dual-task interventions may help participants to automate a task, to focus on other tasks and consequently, to free the individual's processing capacity. After dual-task exercise more attention is available to process external information and therefore to react faster on sudden disturbances [32]. The included studies showed that it was generally feasible to apply dual-task interventions, namely combining a traditional physical intervention with a variety of cognitive tasks, in community-dwelling older adults with balance impairments. During the selection stage of this review, numerous studies were identified studying the dual-task abilities of older adults, though only six studies were found which integrated the method of dual-tasking in a program designed to improve physical functioning. Two studies included relatively simple cognitive tasks like computing or reciting poems. Both studies showed no improvements in physical functioning that were clearly attributable to the dual-task intervention.

Using dual-task exercises with variable-priority or using a complex stepping task may both be closer to real-life conditions as compared to computing while walking. The studies of Silsupadol et al. [60-62] and Shigematsu et al. [58,59] applied a more challenging way of attentional demanding tasks, and, presumably thus, offered advantages in terms of rate of learning compared to more simple cognitive tasks. Results show improvements in functional fitness of lower extremities, balance and gait speed. The latter has been reported as a global indicator of functional performance in older adults and is a good predictor of falls [78].

Shigematsu and colleagues in addition provided a challenging leg exercise which was suggested to enhance neural functions by reducing response latency and by effectively recruiting postural muscles resulting in an improving of the interpretation of sensory information. Caution seems to be indicated in relation to the transfer effects of this form of training. The pre- and post-tests that were used to assess the effects of training were similar to the cognitive and motor tasks assigned in the interventions. Thus, it cannot be excluded that learning effects were observed instead of real improvements in underlying functional motor skills. From this viewpoint, it is not surprising that participants in dual-task-groups performed better in the post-tests.

The dual-task interventions showed satisfying study quality with a mean of 16.2 points out of a maximum of 22 points. However, the results about the effect of dual-task interventions on physical functioning are controversial. In addition, analogue to the cognitive rehabilitation interventions, the limited number of studies performing dual-task training hampers a generalization of results.

### Computerized interventions

Computerized interventions varied from force platforms with visual biofeedback with relatively simple graphics [52,53,55,57,67,71], to video capture systems that enabled the participant to see her/himself on a screen with attractive and realistic graphics allowing to immerge into the virtual environment [32,46,54,68]. A third set of studies used commercially available video game consoles that combined the simplicity of a weight-shifting training on a platform with the elaborated graphics and motivating games of a video capture system [47-49,64,69]. The study quality of the computerized interventions articles was lower (mean value of 12.8 points out of a maximum of 22 points) as compared with the value of the dual-task studies (16.2 points). In contrast to the dual-task interventions, however, the results of the computerized interventions showed a consistent positive effect on various physical abilities in older adults, patients with traumatic brain injury, and stroke patients. Computerized interventions can also be effectively used in clinical settings. Remarkable is that every study reported that participants were more motivated and compliant with the computerized setting in comparison to conventional physical training programs. Computerized interventions may have engaged people who otherwise would lack interest to undergo a traditional exercise program.

The effects of the video games on cognitive aspects of the participants have, remarkably, not been a specific focus of the various studies. It seems, however, that computer games have the potential to also train cognitive functions [34], including attention and executive functions [22]. Combined with physical exercise a video game or a virtual environment requires sensory-motor function inputs as well as cognitive inputs. The participant is required to orientate her/himself, attend, comprehend, recall, plan and execute appropriate responses to the visual cues provided on the screen [69]. The visual aspect is crucial since with aging, vision remains important in maintaining postural control [79]. Virtual environments have also the potential to specifically include motor learning enhancing features that activate motor areas in the brain [80]. In addition You and colleagues suggest that virtual reality training could induce reorganization of the sensorimotor cortex in chronic patients [81].

As we know from the principles of motor learning, repetition is important for both motor learning and the cortical changes that initiate it. The repeated practice must be linked to incremental success at some task or goal. A computerized intervention constitutes a powerful tool to provide participant repetitive practice, feedback about performance and motivation to endure practice [82]. In addition, it can be adapted based on an individual participant's baseline motor performance and be progressively augmented in task difficulty. Weiss and colleagues [83] suggested that virtual reality platforms provide a number of unique advantages over conventional therapy in trying to achieve rehabilitation goals. First, virtual reality systems provide ecologically valid scenarios that elicit naturalistic movement and behaviors in a safe environment that can be shaped and graded in accordance to the needs and level of ability of the patient engaging in therapy. Secondly, the realism of the virtual environments gives patients the opportunity to explore independently, increasing their sense of autonomy and independence in directing their own therapeutic experience. Thirdly, the controllability of virtual environments allows for consistency in the way therapeutic protocols are delivered and performance recorded, enabling an accurate comparison of a patient's performance over time. Finally, virtual reality systems allow the introduction of "gaming" factors into any scenario to enhance motivation and increase user participation [84]. The use of gaming elements can also be used to take patients' attention away from any pain resulting from their injury or movement. This occurs the more a patient feels involved in an activity and again, allows a higher level of participation in the activity, as the patient is focused on achieving goals within the game [85]. In combination with the benefits of indoor exercises such as safety, independence from weather conditions, this distraction may result in a shift from negative to positive thoughts about exercise [17].

### General methodological considerations

A central element of successful cognitive rehabilitation for older adults should be the design of interventions that either re-activate disused or damaged brain regions, or that compensates for decline in parts of the brain through the activation of compensatory neural reserves [86]. Cognitive activity or stimulation could be a protective factor against the functional losses in old age. Because spatial and temporal characteristics of gait are also associated with distinct brain networks in older adults it can be hypothesized that addressing focal neuronal losses in these networks may represent an important strategy to prevent mobility disability [87]. Interventions should, as previous research suggests, focus thereby on executive functioning processes [9], and in particular on the executive function component divided attention [11], and should include enriched environments that provide physical activities with decision-making opportunities because these are believed to be able to facilitate the development of both motor performance and brain functions [88]. This review encourages the further development of virtual reality interventions, preferably with a randomized control design. Future research that aims to examine the relation between virtual reality environments and improvements in both cognitive and walking skills, and the translation to better performance on selected physical tasks, should design the training content such that the relation between the cognitive and physical skills are more explicitly taken into account, e.g. specific elements of divided attention are integrated in the scenario.

Many of the studies of this review were small and may have lacked statistical power to demonstrate differences, if such differences were present. In addition, the interventions were of relatively short duration and heterogeneous in their design, and most subjects investigated were stroke survivors. Most studies did not specifically focus on physical functioning outcomes from which it is known that these relate to brain functioning. For example, spatial and temporal dual-task cost characteristics of gait are especially associated with divided attention in older adults [11], and are dependent of the nature of the task investigated (preferred versus fast walking).

### Future directions

Future research that aims to examine the relation between improvements in cognitive skills and the translation to better performance on selected physical tasks should take the relation between the cognitive and physical skills into account. The majority of the authors, and above all this holds true for the studies using computerized interventions, does not specifically mention or is even not aware of the potential cognitive aspects of their interventions.

### Limitations

We developed and utilized a structured study protocol to guide our search strategy, study selection, extraction of data and statistical analysis. However, limitations of this review should be noted. First, a publication bias may have been present, as well as a language bias, given that we considered only interventions described in published studies and restricted our search to English, French, and German language publications. Second, as there were only few randomized trials, we also included observational studies, the results of which may be affected by confounding bias due to the absence of random assignment. An additional limitation is that we did not investigate the effect of the interventions in separate populations. One study included in the analysis for example assessed subjects with MCI and dementia [65]. It can very well be argued that the results of cognitive interventions may be expected to be different between cognitively intact, MCI, and demented subjects. This point should be considered in future reviews on this topic.

## Conclusions

The current evidence on the effectiveness of cognitive or motor-cognitive interventions to improve physical functioning in older adults or patients with traumatic brain injury is limited. Yet overall, as the most studies included in this review showed, these interventions can enhance physical functioning. The heterogeneity of the studies published so far does not allow defining the training methodology with the greatest effectiveness. This review nevertheless provides important foundational information in order to encourage further development of novel cognitive or cognitive-motor interventions, preferably with a randomized control design. Future research that aims to examine the relation between improvements in cognitive skills and the translation to better performance on selected physical tasks should take the relation between the cognitive and physical skills into account. The majority of the authors, and above all this holds true for the studies using the computerized design, does not specifically mention or is even not aware of the potential cognitive aspects of their interventions.

## Competing interests

The authors declare that they have no competing interests.

## Authors' contributions

Conception and design: GP, EDdB; screening: GP, EDdB, data abstraction: GP, EDdB; data interpretation: GP, EDdB; manuscript drafting: GP, EDdB, PW; KM, GP, EDdB and PW critically revised the manuscript for its content and approved its final version.

## Pre-publication history

The pre-publication history for this paper can be accessed here:

http://www.biomedcentral.com/1471-2318/11/29/prepub

## Supplementary Material

Additional File 1 Search strategy Click here for file

Additional File 2 Assessment of methodological quality Click here for file
