# Supplementary material for: Cognitive and cognitive-motor interventions affecting physical functioning: A systematic review
Source: BMC Geriatr. 2011 Jun 8;11:29. doi: 10.1186/1471-2318-11-29 (PMC3147016; doi:10.1186/1471-2318-11-29)
Supplement: Additional File 1 — Search strategy [file 1471-2318-11-29-S1.DOC]

**Additional File 1 – Search strategy**

| **Area** | **Search terms** |
| --- | --- |
|  |  |
| ***Population*** | (aging or aged or elder*)  exp Aged  exp Brain Injuries  ((injur* or trauma*) adj2 (brain or head or craniocerebral)) |
| ***Outcomes/ Physical aspect*** | ((quantify* or measure* or assess* or investigat* or examin* or evaluat*) adj5 (gait or walk* or balance or movement or mobility or posture or “motor function” or “physical functioning” or frailty)  (balance adj3 (training or impair* or effect*))  postural balance, gait, walking  Accidental Falls [Prevention and Control]  equilibrium, postur* |
| ***Intervention*** | (strateg* adj3 (training or learning or cognit* or metacognit*))  ((cognit* or metacognit*) adj3 (intervention or rehabilitation or task or strateg* or therap*))  (goal adj1 (setting or planning or attain* or achiev* or direct* or orient* or manag*))  (self adj3 (talk or evaluat*))  (self adj3 (awareness or monitoring or control or instruction or regulation))  (“executive functions” or metacognition or awareness or “problem solving” or metamemory or attention)  biofeedback, user-computer interface  (action game* or virtual reality or video game*)  (computerized adj10 training)  (computer* adj10 biofeedback)  ((cognitive or dual) adj5 task  “self-directed learning”, “task performance”  mental imager* |
