# Supplementary material for: Cognitive and cognitive-motor interventions affecting physical functioning: A systematic review
Source: BMC Geriatr. 2011 Jun 8;11:29. doi: 10.1186/1471-2318-11-29 (PMC3147016; doi:10.1186/1471-2318-11-29)
Supplement: Additional File 2 — Assessment of methodological quality [file 1471-2318-11-29-S2.DOC]

**Additional File 2: Assessment of methodological quality**

|  |  | **Reporting** | | | | | | | |  | **External validity** | |  | **Internal validity**  **Bias** | | | | |  | **Internal validity**  **Confounding** | | | |  |  |  |
| --- | --- | --- | --- | --- | --- | --- | --- | --- | --- | --- | --- | --- | --- | --- | --- | --- | --- | --- | --- | --- | --- | --- | --- | --- | --- | --- |
|  | Study | Hypothesis/aim/objective clearly described | Main outcomes clearly described in the introduction or methods section | Participants’ characteristics clearly described | Intervention clearly described | Distribution of principal confounders clearly described | Main findings of the study clearly described | Estimation of random variability for main outcomes provided | Actual probability values reported (e.g. 0.035 rather than <0.05) | **Total** (max: 9) | Subjects asked to participate representative for the entire population from which they were derived | Subjects prepared to participate representative of the entire population from which they were recruited | **Total** (max 2) | Attempt to blind those measuring the main outcomes | No retrospective unplanned subgroup analyses | Statistical tests used to assess the main outcomes appropriate | Compliance with intervention/s reliable | Main outcome measures accurate (valid and reliable) | **Total** (max: 5) | Patients in different intervention groups | Patients recruited over the same period of time | Study subjects randomized to intervention groups | Adequate adjustment for confounding | **Total** (max: 4) | **Power** (max: 2) | **Total**  **Score**  (max:  22) |
| **COG** | Batson et al [34] | 1 | 1 | 1 | 1 | 0 | 1 | 0 | 0 | **5** | 0 | 0 | **0** | 0 | 1 | 1 | 1 | 1 | **4** | 1 | 1 | 1 | 0 | **3** | 0 | **12** |
|  | Dunsky et al [41] | 1 | 1 | 1 | 1 | 0 | 1 | 1 | 0 | **6** | 1 | 1 | **2** | 0 | 1 | 1 | 1 | 1 | **4** | 0 | 0 | 0 | 0 | **0** | 2 | **14** |
|  | Hamel & Lajoie [42] | 1 | 1 | 1 | 1 | 0 | 0 | 0 | 0 | **4** | 0 | 0 | **0** | 0 | 1 | 1 | 0 | 1 | **3** | 1 | 1 | 1 | 0 | **3** | 0* | **10** |
| **DT** | Shigematsu et al [49] | 1 | 1 | 1 | 1 | 2 | 1 | 1 | 1 | **9** | 1 | 1 | **2** | 1 | 1 | 1 | 1 | 1 | **5** | 1 | 1 | 1 | 1 | **4** | 2 | **22** |
|  | Shigematsu et al [50] | 1 | 1 | 1 | 1 | 2 | 1 | 1 | 1 | **9** | 0 | 0 | **0** | 0 | 1 | 1 | 1 | 1 | **4** | 1 | 1 | 1 | 1 | **4** | 2 | **19** |
|  | Silsupadol et al [53] | 1 | 1 | 1 | 1 | 0 | 1 | 0 | 0 | **5** | 0 | 0 | **0** | 0 | 1 | 0 | 1 | 1 | **3** | 0 | 0 | 0 | 0 | **0** | 0 | **8** |
|  | Silsupadol et al [51,52] | 1 | 1 | 1 | 1 | 2 | 1 | 1 | 1 | **9** | 0 | 0 | **0** | 1 | 1 | 1 | 1 | 1 | **5** | 1 | 1 | 1 | 1 | **4** | 0 | **18** |
|  | Vaillant et al [57] | 1 | 1 | 1 | 1 | 0 | 1 | 1 | 1 | **7** | 1 | 1 | **2** | 0 | 1 | 1 | 1 | 0 | **3** | 1 | 1 | 1 | 0 | **3** | 0 | **15** |
|  | You et al [61] | 1 | 1 | 1 | 1 | 2 | 1 | 1 | 1 | **9** | 0 | 0 | **0** | 0 | 1 | 1 | 0 | 1 | **3** | 1 | 1 | 1 | 0 | **3** | 0 | **15** |
| **COM** | Bisson et al [35] | 1 | 1 | 1 | 1 | 0 | 1 | 0 | 0 | **5** | 1 | 1 | **2** | 0 | 1 | 1 | 1 | 1 | **4** | 1 | 1 | 0 | 0 | **2** | 0* | **13** |
|  | Broeren et al [36] | 1 | 1 | 1 | 1 | 1 | 1 | 1 | 0 | **7** | 0 | 0 | **0** | 0 | 1 | 0 | 0 | 1 | **2** | 0 | 0 | 0 | 0 | **0** | 0 | **9** |
|  | Buccello-Stout et al [37] | 1 | 1 | 1 | 1 | 1 | 1 | 0 | 0 | **6** | 1 | 0 | **1** | 0 | 1 | 1 | 1 | 1 | **4** | 1 | 1 | 1 | 0 | **3** | 0* | **14** |
|  | Clark et al [38] | 1 | 1 | 0 | 1 | 0 | 1 | 0 | 0 | **4** | 0 | 0 | **0** | 0 | 1 | 0 | 1 | 1 | **3** | 0 | 0 | 0 | 0 | **0** | 0 | **7** |
|  | de Bruin et al [39] | 1 | 1 | 1 | 1 | 1 | 1 | 1 | 1 | **8** | 1 | 0 | **1** | 0 | 1 | 1 | 1 | 1 | **4** | 1 | 1 | 0 | 1 | **3** | 0 | **16** |
|  | Deutsch et al [40] | 1 | 1 | 1 | 1 | 0 | 1 | 0 | 0 | **5** | 0 | 0 | **0** | 1 | 1 | 0 | 1 | 1 | **4** | 0 | 1 | 1 | 0 | **2** | 0 | **11** |
|  | Hatzitaki et al [43] | 1 | 1 | 0 | 1 | 0 | 1 | 1 | 0 | **5** | 1 | 0 | **1** | 0 | 1 | 1 | 0 | 1 | **3** | 1 | 1 | 1 | 0 | **3** | 0 | **12** |
|  | Hinman [44] | 1 | 1 | 0 | 1 | 0 | 1 | 1 | 1 | **6** | 1 | 0 | **1** | 0 | 1 | 1 | 0 | 1 | **3** | 1 | 1 | 1 | 0 | **3** | 0 | **13** |
|  | Jang et al [45] | 1 | 1 | 1 | 1 | 2 | 1 | 1 | 0 | **8** | 0 | 0 | **0** | 1 | 1 | 1 | 1 | 1 | **5** | 1 | 1 | 1 | 1 | **4** | 0 | **17** |
|  | Kerdoncuff et al [62] | 1 | 1 | 1 | 1 | 1 | 1 | 1 | 1 | **8** | 1 | 0 | **1** | 0 | 1 | 1 | 1 | 1 | **4** | 1 | 1 | 1 | 1 | **4** | 0 | **17** |
|  | Lajoie [46] | 1 | 1 | 1 | 1 | 0 | 1 | 1 | 0 | **6** | 0 | 0 | **0** | 0 | 1 | 1 | 0 | 1 | **3** | 1 | 1 | 0 | 0 | **2** | 0* | **11** |
|  | Mumford et al [47] | 1 | 1 | 1 | 1 | 0 | 1 | 0 | 0 | **5** | 0 | 0 | **0** | 0 | 1 | 1 | 1 | 1 | **4** | 0 | 0 | 0 | 0 | **0** | 0 | **9** |
|  | Sackley et al [48] | 1 | 1 | 1 | 1 | 2 | 1 | 1 | 0 | **8** | 1 | 0 | **1** | 1 | 1 | 1 | 1 | 1 | **5** | 1 | 1 | 1 | 1 | **4** | 1 | **19** |
|  | Srivastava et al [54] | 1 | 1 | 1 | 0 | 0 | 1 | 1 | 0 | **5** | 0 | 0 | **0** | 0 | 1 | 1 | 1 | 1 | **4** | 0 | 0 | 0 | 0 | **0** | 0* | **9** |
|  | Sugarman et al [55] | 1 | 1 | 0 | 1 | 0 | 1 | 0 | 0 | **4** | 0 | 0 | **0** | 0 | 1 | 0 | 1 | 1 | **3** | 0 | 0 | 0 | 0 | **0** | 0 | **7** |
|  | Talassi et al [56] | 1 | 1 | 0 | 1 | 1 | 1 | 1 | 1 | **7** | 1 | 0 | **1** | 0 | 1 | 1 | 1 | 1 | **4** | 1 | 1 | 0 | 0 | **2** | 0 | **14** |
|  | Wolf et al [58] | 1 | 1 | 1 | 1 | 2 | 1 | 1 | 1 | **9** | 0 | 0 | **0** | 0 | 1 | 1 | 1 | 0 | **3** | 1 | 1 | 1 | 1 | **4** | 0 | **16** |
|  | Yang et al [59] | 1 | 1 | 1 | 1 | 1 | 1 | 1 | 1 | **8** | 1 | 1 | **2** | 1 | 1 | 1 | 1 | 1 | **5** | 1 | 1 | 1 | 0 | **3** | 0 | **18** |
|  | Yong Joo et al [60] | 1 | 1 | 1 | 1 | 0 | 1 | 1 | 1 | **7** | 1 | 1 | **2** | 0 | 1 | 1 | 0 | 1 | **3** | 0 | 0 | 0 | 0 | **0** | 0 | **12** |

**Abbreviations:** COG = Cognitive Rehabilitation Intervention; DT = Dual-task Intervention; COM = Computerized Intervention; * = unable to determine
